# Patterns of unexpected in-hospital deaths: a root cause analysis

**DOI:** 10.1186/1754-9493-5-3

**Published:** 2011-02-11

**Authors:** Lawrence A Lynn, J Paul Curry

**Affiliations:** 1 The Sleep and Breathing Research Institute, Columbus, Ohio USA; 2 Department of Anesthesiology and Perioperative Care, Hoag Memorial Hospital Presbyterian, Newport Beach, CA 92658 USA; 3 Department of Anesthesiology, UCLA David Geffen School of Medicine USA

## Abstract

**Background:**

Respiratory alarm monitoring and rapid response team alerts on hospital general floors are based on detection of simple numeric threshold breaches. Although some uncontrolled observation trials in select patient populations have been encouraging, randomized controlled trials suggest that this simplistic approach may not reduce the unexpected death rate in this complex environment. The purpose of this review is to examine the history and scientific basis for threshold alarms and to compare thresholds with the actual pathophysiologic patterns of evolving death which must be timely detected.

**Methods:**

The Pubmed database was searched for articles relating to methods for triggering rapid response teams and respiratory alarms and these were contrasted with the fundamental timed pathophysiologic patterns of death which evolve due to sepsis, congestive heart failure, pulmonary embolism, hypoventilation, narcotic overdose, and sleep apnea.

**Results:**

In contrast to the simplicity of the numeric threshold breach method of generating alerts, the actual patterns of evolving death are complex and do not share common features until near death. On hospital general floors, unexpected clinical instability leading to death often progresses along three distinct patterns which can be designated as Types I, II and III. Type I is a pattern comprised of hyperventilation compensated respiratory failure typical of congestive heart failure and sepsis. Here, early hyperventilation and respiratory alkalosis can conceal the onset of instability. Type II is the pattern of classic CO2 narcosis. Type III occurs only during sleep and is a pattern of ventilation and SPO2 cycling caused by instability of ventilation and/or upper airway control followed by precipitous and fatal oxygen desaturation if arousal failure is induced by narcotics and/or sedation.

**Conclusion:**

The traditional threshold breach method of detecting instability on hospital wards was not scientifically derived; explaining the failure of threshold based monitoring and rapid response team activation in randomized trials. Furthermore, the thresholds themselves are arbitrary and capricious. There are three common fundamental pathophysiologic patterns of unexpected hospital death. These patterns are too complex for early detection by any unifying numeric threshold. New methods and technologies which detect and identify the actual patterns of evolving death should be investigated.

## Background

Unexpected deaths in hospitals and the complications leading to them often include some form of respiratory failure. The macro and micro system dysfunctions responsible for these unexpected respiratory instabilities have been extensively studied, but there still remains considerable misunderstanding among general care clinicians as to how this all works. Much of the confusion is explained by the beleaguered clinician's susceptibility (aka affinity) for conventional wisdom that promises to reliably simplify or "reduce" the complexity and work so often defining the clinical conditions of their hospitalized patients. Easily tracked physiologic bio-markers with warnings when certain "limits" are breached, e.g. breaching of specific heart rate or respiratory rate thresholds, have for years been thought to reflect the onset of serious clinical instabilities. Alarms on our monitors and the monitors themselves become ever more cosmetically impressive, but still function by warning us of these basic breaches, which in theory should be good for patients if the breaches really do detect clinical instability early, when it can be most effectively treated. Unfortunately, this isn't the case. Recent examination shows our "reductionist" monitoring methods to be substantially over-simplified, capable of reliably detecting clinical instability only much later in its devolution. As a result, stakeholders invested in the status quo are now starting to argue, "Better late than never." This sort of rationale may at first sound reasonable, but thorough analysis should convince you otherwise, exposing it as glib, self serving and seriously flawed by its cost to us all in terms of patient morbidity, mortality, and squandered resources. The late detections being signaled by our monitors are often associated with antecedent false senses of security that actually further delay recognition and rescue otherwise likely to occur through astute and timely clinical observation. Without the monitor, decisions to call for help might well have been made based on clinical signs and symptoms, but these are frequently discounted now because of the reassurance provided by our monitors' silence.

In cases of unexpected onset of respiratory trouble, current detection methods are often ineffective, yet nevertheless have been tenured because of their ease and tradition of use with little modification guided by scientific fact or value measured through patient outcomes. One example (we'll discuss later in detail) making this point is the 90% SPO2 threshold from the pulse oximeter. Many general care clinicians still behave as if this number has magical properties capable of precisely differentiating respiratory stability from failure. Over-simplified concepts like this can be very seductive to harried clinicians inclined to believe in them. Unfortunately, as we soon shall see, any reassurance conveyed by SPO2 values over 90% is often as false as the clinicians' misplaced beliefs.

We will review in detail three very distinct and irreducible clinical paths that make up the majority of unexpected respiratory problems seen in hospitals today. Unless caught early, these paths commonly progress to critical instability and death, with each path so physiologically unique they don't begin sharing any patterns until terminal. What this means regarding the possibility of early detection is that no single threshold bio-marker breach today can identify any of these paths' patterns reasonably near their onsets, but rather only much later if at all. That's bad news for the threshold industry and all of us depending on it, but there is good news. Sequential clinical patterns defining our three instability paths leave behind uniquely distinct "footprints" in real-time. These footprints are built from bio-marker signal composites based on the actual relational (conformational) patterns these bio-markers leave from their changes over time. We will be discussing new alarm processing methods for capturing and recognizing these actual relational patho-physiologic patterns once we've reviewed in depth the inherent weaknesses of our present monitoring paradigm and how it is we've come to this critical juncture in providing patient safety.

Some of the problem has been that reliable information on these unique pattern architectures was until now sequestered to a great extent in niche sub-specialties like sleep medicine. It isn't unusual in healthcare to discover much later important clinical information effectively concealed from the front-line hospital caregiver having to most often deal with its potential co-morbid associations. This front-line lack of understanding is reflected in the unquestioned way we've chosen to monitor our general care patients, and even more by many of our standard-of-care processes when framed in a common sense context of early recognition being the most essential component to successfully reversing any clinical deterioration. Just look at our current management protocols on sepsis. Because sepsis is known to be so lethal, most hospitals today have designated protocol-explicit physiologic criteria that once identified, trigger alarms for immediate rescue deployment. One such criterion is reached when a patient's respiratory rate breaches a threshold set typically at 30 per minute. Yet the definition of sepsis established in 1992 by the American College of Chest Physicians and Society of Critical Care Medicine includes any respiratory rate greater than 20 per minute. Every competent clinician appreciates the deadliness of sepsis and the importance of recognizing it early for a successful resuscitation. This has been called the golden period; when missed, mortality and hospital cost rise dramatically. So how can we argue logically for extending our threshold alarms out past 30 breaths per minute when its defining criterion is so significantly less? Why would clinicians allow such delays in calling for help, knowing their patients' lives depend on early, aggressive therapeutic intervention?

We hope you'll find the following discussion interesting and informative. We expect as well that you may find much of what we have to say provocative, given that we challenge many deeply entrenched cultural beliefs underpinning our hospital general care as it's currently being practiced. For this we don't apologize. Anyone familiar with the day-to-day inner workings of our hospitals knows them to be far from perfect despite the best intentions of many bright, highly skilled clinicians that include our nurses, physicians, and ancillary care providers. This discussion is designed to be straight forward and transparent with its claims well referenced and appropriately illustrated. For all it exposes as being less than optimal, it offers reasonable fixes. Patients deserve what they've in the past taken for granted, optimal management of unexpected clinical change...in short, their safety. This can only happen with both early, accurate detection and timely, competent response. Your advocacy for immediate change being made in our traditional beliefs and choices regarding recognition/response strategy is the only way to assure this safety going forward.

## Methods

The Pubmed database between 1956 and December 15, 2010 was searched for articles relating to methods for triggering rapid response teams and respiratory alarms on hospital wards and for articles pertaining to the fundamental physiologic mechanisms and pathophysiologic patterns of death which evolve due to sepsis, congestive heart failure, pulmonary embolism, hypoventilation, narcotic overdose, and sleep apnea.

## Results

### Overview of Patient Monitoring on the Hospital General Floor

Traditional monitoring on hospital general care floors is based on a 16^th ^century "fire alarm" model where an alarm, such as a bell, begins to sound when a dangerous fire has been detected. In modern hospitals, sets of physiologic parameters (bio-markers) are sampled from patients either continuously or intermittently in order to monitor their clinical conditions for the occurrence of change regarded as dangerous, more specifically any change that coincides with selected threshold breaches thought to represent the onset of clinical instability, i.e. the fire. When these threshold breaches occur, a loud noise may sound or some other signal gets communicated immediately to indicate the onset of this urgency and that help is needed. The actual threshold values being used today are somewhat arbitrary, but generally fall within predictable ranges, e.g. SPO2 of 90-80%, respiratory rates of 30-36/min, or heart rates of 120-140/min [1]. Table 1 presents a various thresholds commonly applied [2].

**Table 1:** Alternative Choices for Numeric Thresholds (used with permission)

VITAL SIGN NUMERIC THRESHOLDS							
	Bradycardia	Tachycardia	Hypotension	Hypertension	Bradypnea	Tachypnea	SPO2
Calzavacca (2008)	<40	>120	<90		<8	>25	<90
Genardi (2008)	<40	>130	<90		<8	>24	
Hravnak (2008)	<40	>140	<80	>200	<8	>36	<85*5 min
Brilli (2007)					<8		<90 (Suppl. O2)
Dacey (2007)	<50*15 min.	>130*15 min			<8	>30	
Halvorsen (2007)	<40	>120	<90		<8	>30	<88 (Suppl. O2)
McFarlan (2007)	<51	>120	<91		<8	>24	<90 (RA) or
							<92 (Suppl. O2)
Offner (2007)	<40	>120	<90		<8	>24	
Sebat (2007)			<90			>19	
Garretson (2006)	<40	>130	<90		<8	>30	<90 (Suppl. O2)
Jones (2005)	<40	>130	<90		<8	>30	<90 (Suppl. O2)
Hillman (2005)	<40	>140	<90		<5	>36	
Tibballs (2005)			Age Index				<90 (Suppl. O2) or
							<60 (Cyanotic HD)
Bellomo (2004)	<40	>130	<90		<8	>30	<90 (Suppl. O2)
DeVita (2004)	<40	>140	<80	>200	<8	>36	<85*5 min
Bellomo (2003)	<40	>130	<90		<8	>30	<90 (Suppl. O2)
Buist (2002)		>130	<90		<6	>30	<90 (Suppl. O2)
Hodgetts (2002)			Weighted				

Once alarms are triggered, most hospitals today have organizational rescue processes in place to deal with them. These commonly involve some variation on a rapid response team (RRT) activation that behaves like a "fire brigade" coming to the rescue of the patient in trouble [3,4]. While this fire alarm and brigade model has been accepted in theory as being a straight forward, reliable way to ensure patient safety [3-5], more recent studies [6,7] and a recent meta-analysis [8] suggest that using these thresholds and rapid response activations may not be nearly as effective as first thought. Assuming this concern has merit, it raises some important questions. Is it the fire alarm (detection arm of the process), the fire brigade (response and treatment arm of the process), or both that are failing to improve outcomes from these kinds of events [9]...and why?

While a lot has been written recently to suggest the problem lies with our relatively new RRT efferent processes (response and treatment arm), evidence can also be found pointing to the fire alarm (detection arm) on our hospital floors as being our weak link. Of the several threshold monitoring applications currently available, the capability and effectiveness of one in particular, threshold based pulse oximetry, has proven to be disappointing in several randomized controlled trials. For example, a 2009 meta-analysis of 22,992 patients studied in randomized trails over the past two decades using threshold based pulse oximetry found no outcome benefit [10]. Included in this review was the 2006 randomized prospective trial [11] of so called "new generation" [12] motion resistant [13] pulse oximeters that we'll take a closer look at later in our discussion.

So what might be the source of the problem if any, and why? Before we begin exploring this, it's important to review some basics. In hospitals today, patients can be found in a variety of unstable states, either from the diseases they have or from the controlled trauma (surgical interventions) they're recovering. In order to assure progressive healing or to best hedge against unanticipated worsening of their clinical conditions, tests are generally ordered in some predictable fashion commensurate with our training to assess either the patients' states of well-being or to possibly expose early trouble and the need for changes in treatment. These tests often yield quantitative results expressed in numeric measurements that sit to one side or the other of some threshold value we've deemed as being a clinically reliable cutoff measurement for whatever bio-marker we happen to be testing. For example, any value equal to or higher than "X" (our deemed threshold) indicates a positive test we can rely on. Test results then confirm or rule out with a high degree of certainty whatever suspicions led to the test being ordered and quite possibly to an action or change in therapy. Traditionally through considerable experimental trial, the behavior of any test under development is either validated or rejected based on its reliability. Reliability is the degree of a particular test's sensitivity (TP/TP+FN) and specificity (TN/FP+TN), both commonly expressed in percentages, and both dependent on a single threshold value selected to serve as the cutoff between what has been designated to be positive and negative (TP = True Positive, TN = True Negative, FP = False Positive, FN = False Negative). Tests from bio-markers that aren't capable of yielding single threshold values that are both reasonably sensitive and specific simultaneously, either are abandoned or combined in series with other tests, the amalgam of which must become reliable in order to be useful.

Tests come in many types and any ongoing monitoring of physiologic bio-markers like heart rate, respiratory rate, blood pressure, SPO2, etc. are examples of one type with all the same rules applying. However, an important difference does exist that unknowingly creates problems. While these bio-marker samplings from patients can be acquired quite easily, often being readily displayed on monitors today in real-time, little has ever been done to either validate their degrees of reliability or for that matter their capability to even be considered reliable. That is to say, we've never ascertained if any of these bio-markers are able to provide us with a threshold value that is simultaneously sensitive and specific for detecting the one thing most important to us: clinical instability in its early evolution, i.e. reasonably close to its onset. Nevertheless, we inherited and have "grandfathered" our deemed physiologic bio-markers through tradition, routinely monitoring for them without a second thought given to these reliability/capability issues, and their importance.

Likewise, the semantics surrounding threshold based reliability is able to be manipulated in ways that can be very misleading. A relatively contemporary example making this point is the SPO2 bio-signal acquired by standard threshold based pulse oximeters on general care floors. When its "high" degrees of sensitivity and specificity are being discussed, the praise refers to the device itself and its accuracy for reliably alarming when a patient's SPO2 has breached a threshold value most commonly set at 90% [2]. The advent of motion tolerant filters and other technologic advances continues to improve the precision of these devices, but all these improvements unfortunately have become confused (even by the FDA oximetry standards committee) with improved "bio" reliability for the detection of what really matters, early respiratory distress [14]. Yet no clinical validation was ever done to establish this. It was selected as little more than a metric friendly, arbitrary guess as to what might possibly constitute a physiologic problem, a choice providing a simplified threshold target for the monitoring industry and the basis for impressive appearing promotional material. However, as we will explain, an occurrence (or lack of occurrence) of a threshold breach at 90% SPO2 is largely meaningless in many clinical settings, rendering these promoted sensitivities and specificities equally meaningless.

Now let's for a moment consider what happens when we monitor patients in terms of our traditional "tests" for respiratory instability, regardless whether it's the pulse oximeter sampling data every 1-2 seconds or the general care nurse checking respiratory rates on their patients once every 8 hours. The clinical nurse is testing these patients by sampling their respiratory rates in specific time sequences and will presumably alarm the appropriate caregiver (and RRT) should that rate go over 30/min or some other similarly protocolized numeric value "deemed" to indicate significant respiratory trouble. Regardless whether the monitor is human or some state-of-the-art technology, the process is identical with its capability dependent on the bio-marker selected to indicate detection of some specific clinical condition or state-of-being, and its reliability determined by the sensitivity and specificity of this bio-marker's threshold value. Today we can show there is a "disconnect" to all this. The considerable complexity of most clinical conditions will render incapable most attempts at threshold monitoring for it. We can show beyond doubt that all our currently "deemed" threshold values used today for exposing respiratory instability fail either to detect its clinical changes early enough or fail to detect it entirely with very few specific exceptions. These faux thresholds have all been derived from over-simplified bio-markers, and much like mathematical fractions that have been improperly reduced, they end up incapable of solving problems, in this case yielding appropriate, timely warnings. In different terms, these over-simplified bio-markers can be described as information fragments unsuited for either comprehensive or early detection of respiratory instability. This points out the importance of respecting the degrees of complexity that define the clinical conditions to which we need alerting.

Threshold monitoring is really the use of data and time "snapshots" to render clinical decisions, such as sounding an alarm. We call this traditional hospital approach, "data/time fragment guided protocolization" (DFGP). Threshold based monitors and their associated alarm processors make up the afferent and efferent components respectively of these DFGP hospital care processes. This simple approach has been the basis for the design of hospital alarm processors since the late 1970s. DFGPs work off basic decision trees, such as "if X then Y" where X is a fragment of data from a single point (or brief segment) of time and Y is an action such as the sounding of an alarm and activation of a rapid response team (RRT). Since both threshold alarm monitors and RRT activation share a common DFGP, and both have performed poorly in prospective clinical trials, the pervasive application of data-time fragment guided protocolization in hospitals is now being questioned [15].

While a DFGP can be quite effective when used to define a straightforward decision protocol based on a single data-time fragment, this generally applies only when the simplified or "reduced" DFGP's data/time fragment accurately reflects the condition to be acted on and stands the test of time as being both capable and reliable within a comprehensive range of clinical settings. One example of an effective DFGP is the application of a sliding insulin dose based on the patient's threshold blood glucose value. However, hospitals, by deploying threshold based pulse oximeters for early detection of respiratory distress on general care floors, extend the application of DFGP inappropriately. Here the highly complex and varied patho-physiologic pattern architectures that comprise unexpected respiratory failure in this environment simply can't be reduced to single, numeric threshold values, i.e. data/time fragments, and remain capable. It's the complexity that spoils these threshold reductions, like a SPO2 breach of 90%, and any hope of there being straightforward relationships between X and Y to define a protocol that then reliably triggers alarms based on such fragments of data and time. No surprise then, that such protocolization attempts to date have failed to improve outcomes in randomized trails [11,16].

These considerations also expose pitfalls that arise from our traditional reliance on receiver operative curve relationships (sensitivity and specificity associations) for providing clinically meaningful endpoints (thresholds) when studying the reliability of systems designed to detect data-time fragments from complex conditions. It's easy to get confused about exactly what it is we think we have reliably tested. We already mentioned "new-generation" motion tolerant pulse oximeters and their excellent sensitivities and specificities (but without any meaningful bio-reliability) for recognizing SPO2 threshold breaches of 90% [13]. This sort of confusion can explain why they failed to yield outcome improvements in a high risk post operative general floor population that was at the time expected to benefit from highly accurate, continuous SPO2 threshold monitoring [11]. Just as evidentially apparent but still largely underappreciated, is that an excellent sensitivity and specificity for detecting one specific cause of respiratory death (the inadvertent loss of the airway in the operating room) does not translate into reliable early detection for the more comprehensive body of clinical patterns that evolve with unexpected respiratory instability on general care floors [7]. Different hospital environments can significantly skew the kinds of clinical events arising from patient populations within them as we will discuss, rendering a DFGP of excellent value in one to be near useless or possibly even worse in another.

For all these reasons, our trust in threshold applications for triggering appropriate RRT rescue is beginning to be reconsidered [9]. The following review offers a thorough historic and physiologic perspective on our "fire alarm" methodology anchored by these traditional data/time fragment guided protocolizations, all within a context of capable, early detection of life-threatening clinical instability on hospital general care floors. It also introduces an alternative approach currently in development and based on capable detection, characterization, quantification and tracking of distinct, relational patho-physiologic pattern changes over time as they arise from three distinctly unique clinical pattern architectures known to frequently end in unexpected hospital death.

### The Patterns of Unexpected Hospital Death (Their associated clinical pattern architectures)

Hopefully by now you are beginning to entertain the possibility of there being fundamental flaws to our threshold based approaches for early detection of clinical instability. We mentioned that attention has now turned to the development of new alarm processors much better at responding to early patho-physiologic pattern changes. These patterns can be read from signals arising out of three distinct clinical instability pattern architectures. We call these pattern architectures, "Patterns of Unexpected Hospital Death" (PUHD). Each PUHD contains elements of respiratory failure and together they define the majority of significant instabilities encountered in the hospital environment leading to unexpected morbidity and mortality. While their patterns are not overly complex, neither can the signals indicating their onset be further reduced to a function of any single or multi-parameter threshold breach, and still remain capable of guiding an early response to the threat they represent. Table 2 defines the clinical pattern architectures of our three PUHD.

**Table 2:** The Three Clinical Pattern Types of Unexpected Hospital Death (PUHD)

*TYPE I*	Hyperventilation Compensated Respiratory Distress *(*e.g. Sepsis, PE, CHF)
	Stable SPO2 with progressively falling PaCO2 eventually yields to slow SPO2 decline (mitigated by respiratory alkalosis) and followed by precipitous SPO2 decline when metabolic acidosis dominates
***TYPE II***	**Progressive Unidirectional Hypoventilation (CO2 Narcosis)**
	Progressive rise in PaCO2 (and etCO2) and fall in SPO2 over 15 minutes to many hours. (Often due to overdosing of narcotics or sedatives)

***TYPE III***	**Sentinel Rapid Airflow/SPO2 Reductions Followed by Precipitous SPO2 Fall**.
	A state of "arousal dependent survival" that occurs only during sleep. Arousal failure allows precipitous hypoxemia during apnea causing terminal arousal arrest.

PUHD Type I is considered the most common mechanism of unexpected death in many hospitals, but PUHD Types II and III are the most feared because they have been traditionally associated with clinical error, and can be drug induced by overdose or discovered after the fact in patients with hidden vulnerabilities (such as pre-existing sleep apnea).

Conventional wisdom appreciates only the first two of these three clinical pattern architectures, and then only in terms of broad generalities with considerable room for misunderstandings regarding their details. Patients experiencing the third and most unappreciated PUHD (associated most commonly with sleep apnea), have routinely had their clinical courses misattributed to one of the first two, once the instability has been detected late or after the fact in an outcome analysis [17]. These diagnostic misattributions have perpetuated misunderstandings among well intended clinicians from many specialties including Anesthesiology, Pulmonary Medicine, Critical Care, and Palliative Medicine. Only recently have sufficient case reports been published to unequivocally establish our third pattern's importance. We'll begin our discussion with arguably the most recognizable of the three, and certainly the PUHD most likely to be recalled by clinicians when asked to think back on challenging cases involving unexpected respiratory failure they've had to manage during their careers.

### Type I Pattern of Unexpected Hospital Death (PUHD) (hyperventilation compensated respiratory failure)

**Figure 1:**
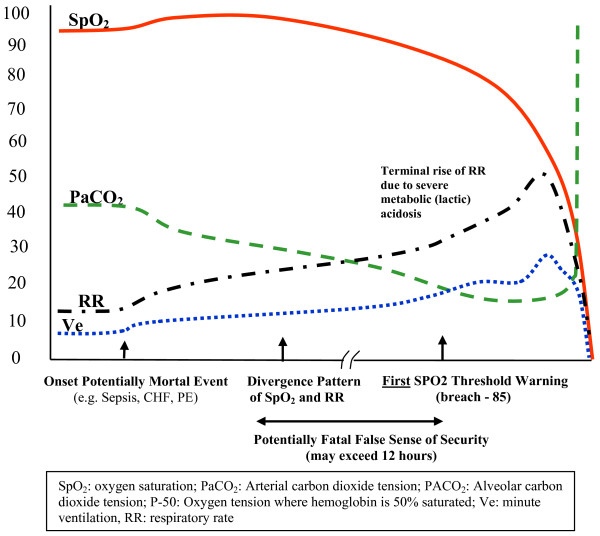
**Type I Pattern of Unexpected Hospital Death (e.g. Sepsis, CHF, PE)**. (Values on Y axis are for reference, actual values for each parameter will vary significantly).

This pattern architecture reflects a clinically evolving process associated with microcirculatory failure induced by such common conditions as CHF, sepsis, and pulmonary embolism to name a few. For this reason it represents the most familiar general process that devolves unexpectedly to death occurring in our hospitals today. Its provenance can be described as being a physiologic response to an earliest posed metabolic and hypoxic threat, beginning with hyperventilation, primary respiratory alkalosis, and an increase in blood oxygen stores. Isolated respiratory alkalosis (RA) has been shown to be the most common early clinical manifestation in patients with sepsis, [18-20], CHF [21], and pulmonary embolism [22]. It characteristically evolves into a persistent alkalosis despite subsequent progressive increases in anion gap and lactic acid levels, well before the development of dominate metabolic acidosis (MA). In fact, during evolving sepsis the brain responds to endotoxin with a rise in minute ventilation even before lung water augments the central ventilation drive [23,24]. These early, incremental steps (initial isolated RA followed by mixed RA and MA, followed by dominate MA) have also been clearly demonstrated in early animal sepsis models [24-26]. The typical progression of Type I PUHD is shown in figure 1.

Predictably, any clinical pattern that progresses from initial dominate respiratory alkalosis should demonstrate elevated respiratory rates associated with the rising minute ventilation (Ve) (see figure 1). However, the very high respiratory rate thresholds (above 30/min) that are customarily used to trigger RRT activations [1,2], have been found to occur most commonly in non survivors [27] with no evidence showing these high respiratory thresholds are breached early in sepsis or any of the other conditions producing Type I PUHD.

Very high respiratory rates (above 30/min), like high lactate levels [28], are likely to assist detection only when severe metabolic acidosis, a late Type I PUHD manifestation, enters the picture (see the late phase of figure 1). They are best considered markers of severity and diagnostic delay [29] rather than useful warnings for early disease. In fact, since any rise in minute ventilation is comprised of both increases in respiratory rate and tidal volume, the use of the respiratory rate alone can never by itself reliably provide a determination of the degree of augmentation of a ventilation response during Type I PUHD. This early phase does have the PaCO2 falling, but the PaO2 and SPO2 most often remain unchanged [30-33] in response to the hyperventilation. While etCO2 closely follows PaCO2 in pediatric patients [34], the relationship is not always straightforward in adults [35,36]. Changes in etCO2 early in Type I PUHD in non-intubated patients on hospital general care floors have not as yet been described.

As Type I PUHD progresses beyond its initial, isolated hyperventilation phase, microcirculatory failure (an early pre-curser and component of shock) develops [37]. In the lungs, this microcirculatory failure causes a progressive decline in the efficiency of gas exchange [38,39]. However, because the patient already has significantly increased minute ventilation, the O2 "seen" by the oximeter's fingertip SPO2 sensor often appears unchanged and stable since greater volumes of oxygen are now being ventilated into the lungs to maintain normal blood oxygen levels. The appearance of physiologic stability, as reflected by these "stable" SPO2 values, persists as this compensation continues to mount, with heightened, ever increasing O2 flux though the trachea/bronchioles required to maintain it. What is actually being seen by the bedside clinician is pseudo-stability created from the compensatory ventilatory change, unfortunately often mistaken for true stability from not understanding the physiologic processes that drive a pulse oximeter's output. Put another way, this very common scenario depicts pulse oximetry's insensitivity for detecting Type I PUHD early (the golden period) when it is most likely to respond to aggressive management. This likewise explains the well intended but ineffective orders most often found at this stage along a devolving Type I process (stepwise increases in the amount of supplemental oxygen delivered), only stalling the delivery of more appropriate therapies.

Eventually, despite these ever increasing tracheal O2 fluxes, microcirculatory failure in the lungs does bring about a fall in PaO2 [40], but this too remains hidden from the pulse oximeter's SPO2 sensor by a contemporaneous rise in pH induced from hyperventilation. Hyperventilation can perpetuate SPO2 values well above 90% regardless a falling PaO2 because of compensatory molecular changes in the hemoglobin molecule caused by the respiratory alkalosis [41]. It's precisely these early, compensatory physiologic changes in particular, and the oximetry patterns from Type I PUHD in general, that can fool clinicians into mistakenly believing these patients aren't in trouble. Threshold pulse oximetry alarms set at SPO2 90% provide this false sense of security when relied on as the "go to" detector for early respiratory distress, made that much worse if even lower thresholds for alarming (e.g. 80%-85%) are being selected to counter what is currently viewed as a "false positive" alarm problem, another misattribution issue we'll be discussing a little later.

As the Type I pattern continues evolving (often over 0.5-48 hours), additional microcirculatory failure and an increase in endogenous catecholamine release cause a progressive lactic acidosis [33], which becomes ever more "naked" through the loss of blood bicarbonate and other endogenous buffers, so that now the stability of the patient's pH is totally dependent on a low PaCO2 maintained through an exhaustive, persistent hyperventilatory response. At this juncture the patient has become highly vulnerable to any interventions that might attenuate an ever more fragile hyperventilation compensatory drive (e.g. narcotic administration), which without standby ventilatory support is often lethal. The conformational change in the hemoglobin molecule (left shift of the oxyhemoglobin disassociation curve) and the high tracheal/bronchiole O2 flux continues to protect the SPO2 from falling, but the sustainable compensatory reserve is rapidly exhausted by an ever higher minute ventilation demand and further depletion of physiologic buffers. Any remaining signs of stability cannot be sustained for long, with fulminate decompensation actuated even earlier by the CHF, septic process, or the PE. During its final sequence, as the patient fatigues [42] or as the CHF, PE, or sepsis advances, pH begins to decline. This initial fall in pH then reverses the conformational change of the hemoglobin molecule shifting the oxyhemoglobin curve to the right [33,41], which activates an accelerating, vicious cycle that includes precipitous declines in both pH and SPO2, eventually producing a terminal collapse in ventilation with concurrent, abrupt rises in PaCO2.

The associated clinical signals of the Type I PUHD have the SPO2 falling only quite late, but when it does, it falls precipitously. By the time the SPO2 breaches a threshold value like 90% or lower, the golden period has been missed and it is often far too late to intervene without utilizing maximum critical care resources and accepting a much greater probability for hospital morbidity and mortality. This can also explain a common but incorrect perception held by many clinicians relying on alarms from threshold monitoring. They often misinterpret the fulminate signs and symptoms from these patients as having developed precipitously, with the accompanying respiratory work believed to be derived solely as contemporaneous compensation for the associated metabolic acidosis. The truth differs, and it's not unusual to discover through careful hindsight inspections of the clinical observations made during these devolving scenarios that signals of progressive clinical distress had been present for hours and sometimes even days before any recognition and/or definitive action being taken. Likewise, as we mentioned, these early signals don't often include significant SPO2 change, the unfavorable PaO2 changes remaining concealed from oximeters because of our compensatory adaptations, e.g. respiratory alkalosis and hemoglobin affinity. The patterns typically seen are imparted from the body's design to deliver oxygen first and foremost when the going gets tough. When it simply can't go on any further, the hydrogen ion stability, conformational changes of the hemoglobin molecule, and fall in SPO2 combine to produce a resounding state of total respiratory collapse that's difficult to miss, with death following quickly. The message is hopefully clear. Threshold pulse oximetry does little to detect early Type I PUHD. If anything it works against the clinician, falsely reassuring when otherwise aggressive strategies might have been initiated at the onset of subtle respiratory complaints.

To summarize, this unique Type I process starts first with clinical patterns being signaled from a rising minute ventilation and a falling PaCO2, then a slow fall in SPO2, a more rapid rise in minute ventilation (and at this point a severe rise in respiratory rate and marked additional fall in PaCO2), followed then by a rapid drop in SPO2 (often only now passing through the SPO2 alarm threshold), and finally (terminally) a fall in respiratory rate and rise in PaCO2. If supplemental oxygen is provided as is often the case, e.g. prophylactic or progressive nasal cannula O2 to manage vague, early symptoms associated with Type I PUHD, the SPO2 can remain stable even closer to the death point, prolonging the false senses of security. Legitimate signal patterns capable of revealing this Type I process early would have to come from a constellation of time integrated changes that reflect steady increases in minute ventilation (airway O2 flux) with consistent blood gas compensations and subtle, early hydrogen buffer depletion, all occurring without significant change taking place in SPO2 observations (diagnosis by exclusion). This requires a series of complex data fragments to be collected and aligned over time, all irreducible to the thresholds currently being used on hospital floors. The sooner we acknowledge this, the sooner we'll be able to salvage these patients early when mortality and morbidity can be reliably avoided.

So exactly how did the magical 90% SPO2 threshold concept gain its advantage in hospital floor care, while ever since misguiding so many well intended general care clinicians? How can so many believe 'above 90% SPO2s' are the sine qua non of respiratory robustness and breaches of 90% the first indication of trouble? To understand this well, we'll have to look at more than just the science behind respiratory physiology. We need to detour momentarily and embrace our psyche, taking a moment to explore the human condition from an evolutionary psychological perspective before easing ourselves back onto more familiar ground.

### Monitoring Revisited With a Twist

We'd suggested at the very beginning of our discussion that all of us share natural affinities (aka biases). There are any number of these biases wired into our DNA, such as our general need and liking to have the vast, complex information that's constantly barraging our senses reduced into more easily interpretable patterns (even if possible bimodal "on-off" or "safe-unsafe" signals) with one important stipulation: that we believe and trust (aka "can be certain") these reductions are accurate, reliable, and useful representations of the complexity from which they come. In fact, "being certain" is another example of one of our biases. Evolutionary bio-psychologists recognize these affinities as being innate, functionally adaptationist products of natural selection that enhance our probability for survival, or more accurately the survival of our chromosomes [43-45]. With this in mind, the short answer explaining our penchant for simple thresholds, such as our magical 90% SPO2 detector, comes only in small part from it being the way we've been taught. We are much more likely attracted to trusting these thresholds because of their extremely easy-to-follow "rules of engagement," without which we would be left vulnerable to constant worry, stress, and indecision managing our sick in need within the confines of our chaotic hospital environments. Combining anything we've been taught with our natural affinities for simplification, certainty, and summary judgment, the odds soar for the resulting behaviors ending up culturally ensconced. Unfortunately, these particular behaviors have inadvertently erased our objectivity and made it extremely difficult for us to detect early patterns of unexpected hospital death in many of our clinical environments, a point that should now be obvious from the details we reviewed regarding Type I PUHD. Type II and Type III PUHD present their own uniquely challenging problems associated with adapting our simplistic threshold concepts for their early detection, but we'll discuss them after a bit more on just how to date we've gotten so far off track regarding our belief in the capability of threshold based technologies. Our decades' old culture of threshold monitoring has created its own worldwide infrastructure of monitoring scientists, industrial designers, standards committees, and marketing teams. Make no mistake about it. This is very much about big business and big business behaviors. We'll take a moment now to penetrate its "veil of science."

Fundamental to the science and efficacy of threshold monitoring is the initial choice of a discreet numeric value within an available set of parametric values to be used to represent the very best indication that something important either has, or is about to occur. This sounds simple enough to do until you begin in earnest exploring the essential characteristics this value must have. Knowing for certain an exact value that defines an important change, like any number over 20 for breaths per minute with sepsis, won't necessarily work. It's not because it isn't the most sensitive number for the job; it's because it is a very non-specific number [46]. This simply means that if we were to use this as our threshold cutoff value, we'll certainly capture nearly all early septic patients as intended, but we'll also drag lots of other patients without it along with them, patients who have other, often innocent reasons for breathing more quickly. The additional burden these other patients add from their consumption of a fixed amount of very valuable rescue resources would significantly mitigate the effectiveness of the response to the true event. Arguably even more detrimental is the amount of rescue response fatigue produced by these added false positive alarms having to be answered, i.e. the "Cry wolf" effect.

Despite the importance of the pivotal positions of threshold values chosen by the monitoring industry, the actual threshold numbers suggested have been traditionally arbitrary and often capricious. This exposes threshold "science" as conjecture, exemplified by the recent publication of a matrix of published chosen thresholds [2], which allow proponents of threshold monitoring to choose almost any range of trigger values they wish. Whether capriciously derived or not, for any single threshold value to be clinically reliable for monitoring, the chosen value must be both highly sensitive and highly specific simultaneously regarding all the important clinical processes needing detections and interventions. We have already pointed out that the solution provided by the industry and other active proponents of thresholds, is to make the target simple and to cite sensitivity and specificity as a function of how accurately the monitor derives this arbitrary target value. For the rest of us, this solution should no longer be acceptable because it skirts the central issue, that being there are no single threshold values within our vital sign parameters routinely monitored on hospital floors today that are both highly sensitive and highly specific for any of the early patterns of unexpected hospital death. The typical numeric values we've accepted and worked with for decades vary widely, but only become consistently very sensitive and specific together when instability has long established itself (late) in two of our three PUHD, while never becoming specific until terminal in our third. The gap between late and early recognitions for each pattern architectural type is filled with needless morbidity, mortality, and cost. This issue constitutes a huge problem that has set some of us to working diligently for innovative solutions beyond our "tenured" monitoring traditions, while others continue fighting to preserve the worn and ineffective status quo.

We'll make this clearer by revisiting our sepsis example. An adult respiratory rate in breaths per minute of any number above 20 has been defined as a component of the systemic inflammatory response syndrome accompanying sepsis. While the 20 value chosen as our threshold serves us well regarding sensitivity, it's simultaneously very non-specific. Using it as your alarm threshold would result in its alarming for large numbers of patients all the time. Granted, among them you'd capture all your early septic patients, but you'd also be including many non-septic patients. Most of these would have benign conditions that would then be labeled as false positives, but only after fatiguing the nurses and physicians on the floors and consuming inordinate amounts of fixed resources. You might say it's worth it, and in theory that's perhaps superficially admirable, but extremely impractical and disruptive. Working effectively in a chaotic general care environment is difficult enough without heaping on the enormous distraction set in play by numerous false alarms. The added waste of energy, time, and resources combine to make the environment exponentially less safe, far outweighing the noble premise behind rescuing sepsis early at all costs. Only when the respiratory rate breaches 30 breaths per minute does that particular threshold become reliable, meaning both sensitive and specific enough for rescue to be deployed sensibly, but unfortunately now at much later stages within the devolving disease process. The value '30 BrPM' here is a value of compromise, hinged on the misleading premise "better late than never," and established to serve both the greater good and the individual patient simultaneously. It reflects a process that should get at best a grade of C-, a process to be merely tolerated while we figure out ways to deliver an A+ solution. Instead, the process has been culturally transformed into one of our gold standards. Similarly, and we pointed this out earlier, the 90% SPO2 pulse oximeter threshold is also a C- value of compromise, both sensitive and specific only for late Type I respiratory instability, not early when Type I has its best chance of being managed successfully. So how exactly was the magical 90% SPO2 threshold conjured up? The answer can be found in its provenance.

### The History of Threshold Monitoring on Hospital General Care Floors

Simple warning electrocardiographic monitoring systems which triggered alarms in response to high or low heart rates were developed in the early 1960 s [47], and these represented some of our earliest threshold monitors. An arbitrary value from high or low QRS complex occurrence rates (heart rate) was selected and an alarm would sound if this was breached. Low heart rates below 50 beats per minute and high values above 120 were commonly selected. When the pulse oximeter was introduced in the early 1980 s this same threshold based approach was applied with 90% arbitrarily made the SPO2 cutoff value. This choice was largely cultural, reflecting a general scientific goal at the time to go metric. Certainly the value derived by subtracting 10 from 100 nicely aligned with that vision. Using numeric increments of 10 are technically easier to work with, even if the number isn't being derived through "bio"-scientific inquiry. The idea that nature conveniently provides us with this "ideally" rounded threshold set precisely at the 90% saturation "knee" of our oxyhemoglobin dissociation curve was an afterthought that makes little sense for many reasons. Perhaps the most important of them is the dissociation curve's ability to shift both right and left through sequences within our patterns of evolving death, rendering the concept of the relationship of the "knee" to a fixed SPO2 threshold meaningless in clinical crisis. However, as already discussed, we like our complex concepts simplified, and this 90% SPO2 "knee" featured splendidly as an industry marketing ploy, at once sounding both impressively sophisticated and easy... then shortly thereafter becoming enhanced remarkably through a stroke of serendipity.

Breaking into hospital based healthcare markets has never been easy, but a path was cleared for oximetry's entry into surgical operating suites (ORs). At the time of its introduction, the most feared mishap arising from this environment was an undetected loss of airway. Unique to operating rooms and special care units is a prevailing risk for airway loss because of the frequent use of general anesthesia and mechanical ventilation. In these controlled environments there's little else that causes an otherwise stable patient receiving supplemental oxygen, as most do, to suddenly drop from expected hemoglobin saturations to 90% or lower. What's more, any desaturation breaching 90% SPO2 with an anesthesiologist or critical care physician at that bedside leaves ample time for a properly deployed rescue without any harm coming to the patient. Said another way, these environments just happen to be uniquely suitable for this particular threshold monitor because unlike our Type I example on the general care floor, here a 90% SPO2 threshold breach is both extraordinarily sensitive and specific for indicating the near onset of the one lethal problem most likely to occur, and it triggers into immediate action its precise correction. It was this quite unusual alignment of early (near onset) capability and reliability with the efferent, nearby rescuer that formed an ideal DFGP (Data-time Fragment Guided Protocolization)...and it catapulted the perceived value of pulse oximetry and its reputation for enhancing patient safety through the stratosphere, but unfortunately without a contemporary appreciation for why it was bound to succeed in this environment, but not in others.

An unparalleled enthusiasm for pulse oximetry in the operating room propelled its nearly instant migration into special care units like perioperative recovery rooms and critical care units, where similar risk for airway loss and the immediate availability of specialty trained, competent airway experts also coexisted. Pulse oximetry's life-saving reputation continued to swell, and with it the single minded opinions of those clinicians used to its application in these specific locations. Well-intended anesthesiologists and critical care physicians, because of their deep but narrowed expertise, couldn't be faulted for wanting to enhance patient safety everywhere, but had sparse experience with the patho-physiologies of true sleep related upper airway disturbances, or for that matter with most any general care issues being managed on the hospital floors. Their misplaced enthusiasm for deeming pulse oximetry's SPO2 as our next "vital sign" for all patient environments, along with its magical 90% threshold, would immediately open Pandora's Box. We've seen how ill-suited the 90% SPO2 threshold is for detecting early Type I PUHD. It was also becoming evident by the late 1990 s that an untoward clinical "attitude" was emerging on the floors; that false senses of security were being encouraged by the 90% SPO2 threshold, not just delaying early clinical interventions, but promoting actual discounting of fairly obvious clinical symptoms. In previous decades a nurse or house officer would likely have proceeded with a comprehensive evaluation of any complaints of dyspnea, probably ordering at minimum a blood gas and chest radiograph. Now, reassured by accompanying saturations in the 90%s, these "reassurances" were being routinely passed along to struggling patients with unintentionally glib remarks like, "You're breathing just fine, your oxygen level is nearly 100%...if you're still short of breath in the morning, I'll call the doctor..." The disservice here to patients should now be obvious, and while perhaps not nearly so obvious, the rejoinder now being heard for maintaining these incapable thresholds, "Better late than never," should be viewed as equally glib and "saturated" with danger.

Beyond these troubling challenges, pulse oximetry's entry into the general care culture in the late 1990 s brought with it a degree of disruption never previously experienced in hospital medicine. The general care floors became instantly inundated with countless alarm events that would for years to come and even today be misdiagnosed, misattributed, and misunderstood. What's worse, some leaders within the oximetry industry today, like carpenters owning only a hammer tend to see everything as a nail, are now suggesting "solutions" that call for adjusting the SPO2 threshold values for our floor care downward, a solution that frankly defies all logic. To fully understand oximetry's disruptive potential and why the technology needs to be overhauled entirely rather than "threshold modified," we need to examine next the details of PUHD Type II and III.

### Type II Pattern of Unexpected Hospital Death (CO2 narcosis)

Well back before the 1950 s [48] and even today, nurses and physicians in training are taught that narcotics produce death through a singular path involving progressive hypoventilation. Perceived as a deteriorating, self propagating process, both the narcotics and a rising PaCO2 contribute to the central depression of ventilatory drive. This "vicious cycle" of narcotic induced central depression begins with its pharmacologic induction of a rise in PaCO2 from neuro-inhibition at the brainstem's ventrolateral medulla pre-Botzinger complex [49,50], that then furthers this central depressive state, ultimately leading to "Carbon Dioxide Intoxication" or "CO2 Narcosis" severe enough to bring on respiratory arrest. Put another way, this represents a distinct form of central nervous system depression resulting in slowed and shallow breathing caused by μ-opioid (μ_2 _subtype) receptor-mediated blockade [51] and possibly also involving active intermediary metabolites of certain narcotics [52], ion trapping in the brain [53], and poor excretion kinetics. Hypoxemia may be evident only terminally in this process if the patient is receiving sufficient supplemental oxygen [54]. As respiratory failure and death devolve through its timeline, supplemental low flow oxygen can hide it and its pathognomonic SPO2 pattern signals entirely from the pulse oximeter until very late [55,56], just as it does with Type I PUHD.

Although recently eclipsed by our nascent realization that "CO2 Narcosis" may not be the most common reason for patients dying silently at night after receiving narcotics, there is no question that this Type II clinical process poses a legitimate threat. Some patients, often hidden in our pre and postoperative populations, are at very high risk for postoperative hypoventilation when given what's considered normal doses of sedatives and/or narcotics. Classic cases of this are seen in adult patients with congenital central hypoventilation syndrome, e.g. those with PHO2XB mutations [57] who can be completely asymptomatic while awake, yet despite normal daytime PaCO2s, exhibit profound hypoventilation responses to sedation and narcotics once asleep. Others at risk include patients with obesity hypoventilation syndrome [58], chest wall deformities, polio sequelae, advanced COPD [59], and severe hypothyroidism [60]. Then there's always the possibility of an accidental narcotic overdose, although our currently piqued awareness regarding medication error has resulted in a wide range of preemptive processes being applied by most hospitals to prevent this.

Our thinking dating back to the 1980 s regarding how we can best monitor for opioid induced respiratory depression called for monitoring the respiratory rate. While some studies have shown that respiratory rate reductions provide a useful indication of ventilatory depression in some patients [61,62], there's also evidence to suggest that it's not quite that simple. Several studies have shown opioid and sedative induced respiratory depression to be frequently associated with reductions in tidal volume and more variable patterns of breathing [63-65]. In fact, the hypoventilation produced by some benzodiazepines may primarily reduce tidal volumes with accompanying increases in respiratory rate [66]. In obese patients, or others with narrow or non-patulous upper airways, tidal volumes may be further reduced through increases in upper airway resistance induced by opioids [67,68], suggesting that any relative reductions in rate and/or tidal volume are likely to be highly variable depending on both patient and drug related factors. Because in so many cases tidal volume may be reduced to a significantly greater extent then respiratory rate, the application of threshold respiratory rate monitoring as the single surrogate marker for opioid-induced respiratory depression can easily provide false senses of security. The addition of pulse oximetry to intermittent or continuous respiratory rate monitoring may be just as inadequate if supplemental oxygen is being provided [55]. Pulse oximetry can be quite sensitive for detecting Type II PUHD once moderate hypercarbic levels are reached if a patient is breathing room air, because the moderate increases in PaCO2 begin to "crowd out" available oxygen at the alveolar interface resulting in relative hypoxia with noticeably declining SPO2 values [69]. However, it remains quite insensitive to even profound hypercarbia when supplemental oxygen is used, as is the current trend in early postoperative management where higher doses of narcotics are more likely to be seen [54,55].

**Figure 2:**
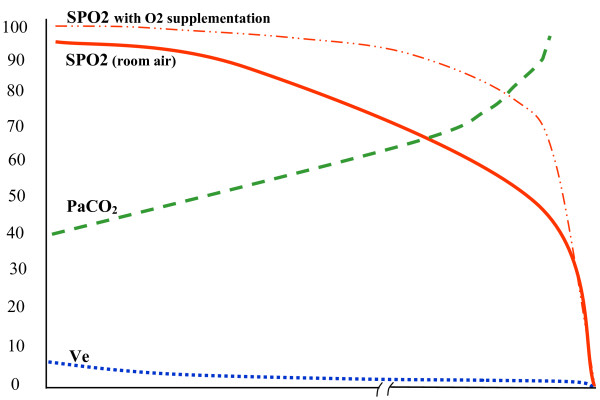
**Type II Pattern of Unexpected Hospital Death (CO2 Narcosis)**.

So in summary, (as illustrated in figure 2) the Type II PUHD comprises first a fall in Ve (the amount of air moved in or out of the lungs per minute) due to progressive falls in tidal volume and/or respiratory rate, both unpredictably variable. This pattern continues to devolve as the body, failing to rid itself of its excess CO2 mounting from inadequate ventilation, begins to suffer from the effects of respiratory acidosis and CO2 narcosis. As the PaCO2 rises higher and higher, it competes with oxygen for space at the alveolar interface, seen reasonably early as a falling SPO2 in patients breathing room air (see figure 2). Because any acute rise in PaCO2 is also associated with falls in pH that shift the oxyhemoglobin disassociation curve to its right, monitored SPO2 declines are magnified by these pH/PaCO2/PaO2 shift effects on the SPO2. However, patients provided with supplemental oxygen can maintain SPO2 values in the 90-100% range with significantly advanced hypercarbia (see figure 2 dotted line), often the first hint of a problem coming from being discovered unarousable in near respiratory arrest or worse. Putting all this in a context of reliability and DFGP capability for early detection and rescue using our magical 90% threshold, pulse oximetry is moderately sensitive only when patients breathe room air, and extremely insensitive when supplemental oxygen is being deployed. Combining sedation scoring and threshold capnometry with pulse oximetry has been advocated by some experts, and this combination appears capable of providing an effective way to detect pure Type II PUHD [70,71], although such additions would be costly and less effective than imagined because of confounding circumstances. What confounds any reliable early detection of Type II patterns (Type I as well) by all threshold applications is our third PUHD, a clinically subtle yet exceedingly common process that only occurs during sleep, and just like the others is not amenable to reliable early detection with any form of threshold monitoring. Likewise, it remains indistinguishable by even the most meticulous sedation scoring. This Type III PUHD, which has been associated with silent, sudden death during sleep, is largely unknown to most clinicians, yet burdens the general care environments with extraordinarily common clinical and statistical mischief regarding any conventional attempts to reliably recognize it and its co-morbid associations. We'll have a look at this third PUHD now.

### Type III Pattern of Unexpected Hospital Death (Repetitive reductions in airflow (RRA) and SPO2 during sleep followed by arousal failure and sudden hypoxic death)

Having just discussed the prevailing belief held for decades (and still being taught in Medical Schools) on the cause of respiratory failure and death induced by narcotics and sedatives, we're now ready to unsettle any certainty and comfort this simplistic belief might provide. A "stand alone" Type II concept has fomented the widely held perception that sedation scoring with threshold alarms, whether from pulse oximeters using SPO2 limits or capnometry using some form of accessible CO2 threshold, can capably and reliably provide early enough detection to allow timely rescue and reversal before harm intervenes. So if otherwise informed clinicians are aware only of Type I and II PUHD, as most today are, then they would believe that setting a pulse oximeter's threshold to 90% would render it capable of detecting advanced Type I instabilities, but would at least allow it to catch Type II reasonably early if their patients aren't receiving supplemental oxygen. It wouldn't be unreasonable for them to also believe this is better than missing both types by not using oximetry at all (discounting our false sense of security issue). They'd likely reason as well that a combination of pulse oximetry and capnometry with frequent sedation score samplings would ensure a safe environment wherever parenteral narcotics were being administered...and they'd be standing in good company because this is exactly the current thinking being fostered by some very bright healthcare leaders as it relates to improving hospital floor safety. However, a well concealed and unappreciated, yet extraordinarily common third PUHD has been making its nefarious presence felt for as long as we've been administering parenteral analgesia in hospitals. It ironically was brought to light by the pulse oximeter's entrée into general care, the irony stemming from a decade's worth of misattributed Type III attenuated (smoothed and unreliable) oximetry signals acquired off patients on hospital floors that have all but made threshold SPO2 monitoring (threshold capnometers are just as vulnerable) near useless for reliable early detections of all three PUHD.

**Figure 3:**
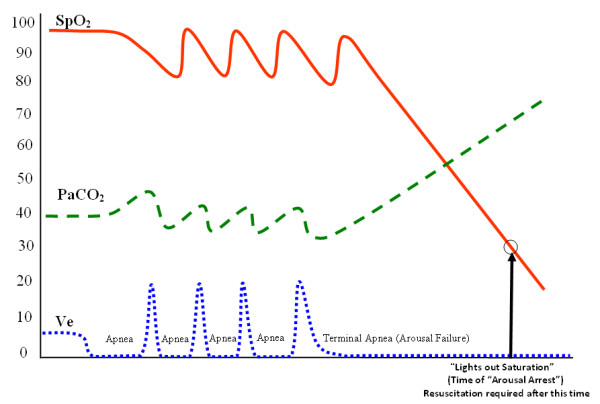
**Type III Pattern of Unexpected Hospital Death (Sleep Apnea with Arousal Failure)**.

Back in 2002, Lofsky [17] described in the malpractice literature a cluster of unexpected hospital deaths involving patients with risk factors for obstructive sleep apnea. These patients had all died in bed and in spite of acceptable dosing of narcotics. Surprisingly, they all shared a unique clinical course that started with being awake, alert, and stable, then sleeping, and then being found dead. The standard dogma prevailed at the time, the thinking being that these deaths occurred because of PUHD Type II hypoventilation (CO2 narcosis), and that the problem could have been avoided simply by using threshold monitoring, either oximetry, capnometry, or both. Now we have good evidence refuting this selective conclusion, showing that subgroups of patients with obstructive sleep apnea, can demonstrate independent, severely delayed arousals in response to their apneas (called occult arousal failure). This condition produces a distinct, high resolution SPO2 respiratory pattern during sleep, which we've named the Type III PUHD (Pattern of Unexpected Hospital Death). It differs from our classic Type II CO2 narcosis process, in that it occurs only during sleep. When awake, patients with profound Type III arousal failure may exhibit no pathognomonic symptoms or signs, or show evidence of any "awake" sedation. In other words, patients with arousal failure are orphaned, remaining completely concealed within our typical pre and postoperative populations. As shown in figure 3, the sentinel instability component of Type III PUHD is induced by sleep apnea in the presence of arousal failure.

**Figure 4:**
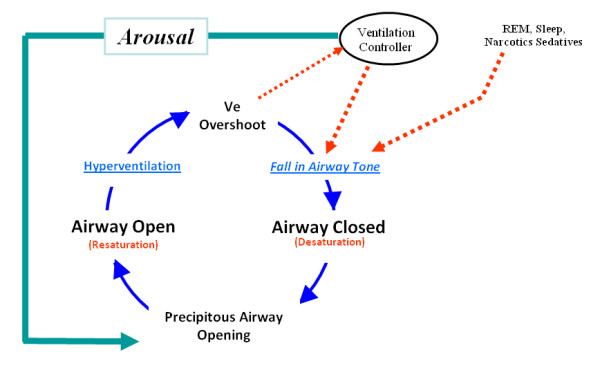
**The Mechanism of the Cycling SPO2 Pattern of the Type III PUHD**.

**Figure 5:**
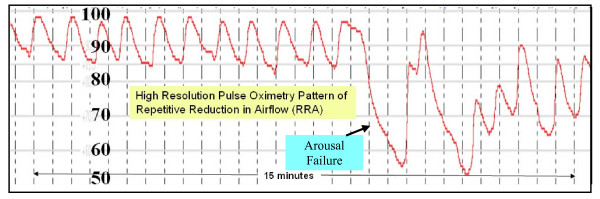
**Type III SPO2 Cycling Pattern ("Sawtooth") Preceding Arousal Failure**.

This Type III pattern architecture is comprised of repetitive reductions in airflow and SPO2 from sleep related cycling collapses of the upper airway [72,73]. This cycling figure 4, collapsing, and reopening of the upper airway produces a typical and very distinctive pattern of signal clusters shown in figure 5, that is reliably acquired only by higher resolution pulse oximetry (unlike many conventional pulse oximetry systems in use on hospital floors today). How this unique SPO2 pattern is produced will be discussed in a moment, but more contemporary bench research on how narcotics interact with our neuroaxis corroborates these important, newly appreciated patterns and their implied threats that include being capable of inducing a de novo form of sleep disordered breathing with repetitive airflow reductions very similar to that found in obstructive sleep apnea populations.

Current research describes narcotics modulating adenosine levels in two critical areas of the brain that influence arousal states, the pontine reticular formation (PRF) and the substantia innominata within the basal forebrain (BF) [74]. Homeostatis between sleep and wakefulness is maintained through interactions among dozens of disparate nuclei spread along the entire neuroaxis. The neural circuits regulating arousal state form a flip-flop switch, in which, at any given time, only sleep or wake-active neurons are firing. Arousal-promoting nuclei (located predominantly in the pons, midbrain, and basal forebrain) and sleep-promoting nuclei (located predominantly in the preoptic hypothalamus) mutually antagonize each other via reciprocal inhibitory connections. Narcotics have been shown to reduce adenosine levels in these critical areas of the brain [75] and this appears to lead to a disrupted sleep architecture, blocking access to rapid eye movement sleep and to the deeper restorative stages of non-rapid eye movement sleep. Doubtlessly, the body of research available today on opioids suggests strongly that narcotics have a much broader effect on brain function than that traditionally attributed to causing Type II PUHD.

As illustrated in figure 4, obstructive sleep apnea can be best understood as a condition where during sleep, one's upper airway collapses and is held closed by vigorous but ineffective respiratory effort (much like trying to suck on a collapsed cellophane straw). Each apnea in a repetitive sequence of cyclic apneas is generally terminated by a micro-arousal that occurs when the person reaches an arousal threshold derived from at least a couple of known components. The arousal then causes brief "overshoot" hyperventilation that drives the PaCO2 below normal. This excessive drop in PaCO2 triggers a fall in one's central drive to breathe and a contemporaneous fall in the central tone of the upper airway. Since the upper airway is already unstable it collapses again, causing the cycle to reenter and self propagate, producing its sentinel pattern of repetitive reductions in airflow and SPO2 [72]. Narcotics [67,68,76], spinal anesthesia [77], sedatives [78] and cycling hypoxemia [79] can increase one's arousal threshold (cause arousal delay), and then death can occur from complete arousal failure (arousal arrest) [80,81]. So the Type III pattern comprises a sentinel grouping of repetitive reductions in airflow (RRA) that produces rapidly cycling SPO2 levels [72] and brain oxygen level reductions [82] with the risk of subsequent, precipitous falls in SPO2 to the point of arousal arrest. Once this occurs, if no intervention is provided immediately, a Type III death will follow suddenly during sleep without warning due to precipitous hypoxemia, and most often without much progressive PaCO2 elevation because of insufficient time for the hypercarbia to develop.

Explanations about the mechanics of self rescue from sleep apneas today include increases in chemical drive that can both excite the upper airway dilating muscles [83] and foment arousal [84]. Arousal has been considered the most essential survival mechanism for these patients in a sense because they sleep in a state of perpetual "arousal dependent survival" with scores of cycling apneas occurring hourly, each of which must be reversed by an arousal to prevent sudden sleeping death. Unfortunately for a subset of these patients, they also have arousal failure [68,78] where their physiologic arousal response to apnea events is delayed significantly, allowing apnea mediated oxygen reductions to progress to severe desaturation levels before arousal reopens their upper airways allowing recovery (figure 5). Anything that might additionally delay these recoveries, e.g. narcotics, creates an extraordinary risk for respiratory arrest [80,81].

Both the cause and incidence of arousal failure in patients with sleep apnea have not been well defined, but it's now known to be not nearly as uncommon as originally thought. It has been postulated that our central arousal systems may acquire arousal failure over time as a function of neural plasticity in response to repetitive exposures to rapid declines in oxygen saturation over many years. As the central arousal system adjusts its response, the arousal itself can become progressively more delayed (much as it would to intermittent loud sounds after years of sleep exposure to the passing of nearby trains). Patients with sleep apnea generally have repetitive exposure to 50 or more episodes of brief hypoxemias every night, and therefore may be at particular risk for acquired arousal failure. This is particularly true of obese patients who on average have more severe cyclic desaturations [85,86]. Their arousal responses to episodes of hypoxemia may become progressively attenuated, requiring ever increasing levels of arterial hypoxemia to induce awakening. If they present for surgery, there is no conventional way to know (unless previously studied) that their arousal system is failing. Whether arousal failure is genetic or acquired over time, patients in perioperative populations with this disorder tolerate long arousal delays, their arterial oxygen saturation values routinely falling into near life threatening ranges many times during sleep, and each time their late arousal recoveries rescuing them from an arousal arrest that can be only seconds away.

One reason arousal delay becomes so critical is that SPO2 is able to fall at very rapid rates during apnea. Many physicians accustomed to witnessing preoxygenated apnea lack a full appreciation for the extremely early and very steep desaturation slopes seen in recumbent, obese patients with low functional residual capacities, breathing room air. With these kinds of apneas, the rate at which arterial oxygen declines is inversely related to the volume of oxygen within the lungs at the apnea's onset, and for many postoperative patients breathing room air this volume can be quite low. In fact, since postoperative functional residual capacity does not have definable lower limits, oxygen desaturation rates may in some cases exceed 1.5% per second with SPO2 falling to critical values with no time for contemporaneous hypercarbia to develop [87]. This is best understood by reading *Wilkinson et.al's *remarkable article [88] explaining this dynamic process. It demonstrates profound rates of SaO2 decline from these rapidly cycling apneas because of multiple airway collapses that occur in sequences typical for sleep apnea combined with arousal failure. Using high resolution pulse oximetry, the components of these patterns can be accurately analyzed and defined [72,82]. With most rapidly falling desaturations, each SPO2's fall is interdicted by an expected arousal that precipitously reopens the airway. But occasionally, in the presence of severe arousal failure, a patient's arterial oxygen saturation can fall to a point where the brain no longer receives sufficient oxygen for central arousal to occur [68,80,81]. This is called the "Lights Out Saturation" (LOS) and happens because our brains are incapable of generating sufficient anaerobic metabolism. We all depend on a continuous supply of oxygen to support our brains' higher functions (such as arousal). If arterial oxygen saturations fall below this critical value where the hemoglobin molecule simply cannot release sufficient oxygen to the brain, EEG slowing occurs promptly and arousal becomes totally suppressed: the "lights are out."

Once the LOS is breached, airway reopening without resuscitation isn't to be expected. Unless discovered quickly, this sleeping brain soon dies. Tragically however, the body remains alive and continues to burn glucose and fat, while producing carbon dioxide that remains trapped because of the collapsed upper airway and absent ventilation, preventing its escape. During this sequence the heart continues to pump ever mounting CO2 stores through an anoxic body. If the patient is discovered now and resuscitation initiated, the immediately drawn blood gas will show the PaCO2 to be quite high, disguising this incident as a Type II event. This accounts for our extended history of so often incorrectly attributing these events to narcotic induced CO2 narcosis. Terminally, ventricular fibrillation, pulseless electrical activity, agonal rhythms, and asystole follow, and the body then dies.

Restated, the major factors capable of inducing such sudden, sleeping deaths (Type III PUHD), are the amalgam of first an underlying (or induced) sleep breathing disorder that requires an arousal response, and with it an added delay in arousal from well intended administrations of sedation or narcotics most likely imposed on some degree of preexisting arousal failure. It's also probable that narcotics are capable of inducing a de novo form of disordered breathing with sleep, independent from its ability to also delay arousal [89]. With both mechanisms in play, the nadir of oxygen saturations brought on by cycling apneas and incomplete recoveries can together produce sufficient cerebral hypoxemia to induce "arousal arrest" [68,80,81]. Then the airway doesn't reopen and without immediate help death follows. This is a very plausible explanation for why these patients are found "dead in bed" with no warning, and why patient controlled analgesia (PCA) may not be as safe as originally advertised, particularly for patients who exhibit these distinct Type III clinical pattern anomalies [76].

In summary, on all general care floors and most certainly on post surgical units, if sleep apnea with its unique state of arousal dependent survival exists but is either unrecognized or is left untreated, only the cycling SPO2 signals acquired off high resolution pulse oximetry can provide sentinel markers for both cyclical apnea occurrences and arousal failure. The administration of narcotics and/or sedatives to patients with preexisting arousal failure can further delay an already failing arousal to the point of arousal arrest. This then completes the pattern architecture associated with Type III PUHD, comprised of sentinel cyclic desaturations followed by precipitous falls in SPO2 to the point of incapacity for self recovery and death. Understanding the importance of these high resolution signal patterns should clearly expose our patients' vulnerability when depending on standard threshold monitoring to assure their safety. The threshold premise relies on a now singular and credulous 1980's and 1990's concept (Type II PUHD) for narcotic induced death. The PCA's (Patient Controlled Analgesia) self medicating design was also based on this oversimplified explanation of death.

If a clinician's understanding is limited to only this Type II explanation, his/her belief that central depression prevents patients from the possibility of self medicating to overdose proportions would logically follow, albeit remain incorrect. Remember, the Type III Pattern was not known about when the PCA was introduced, but we should now be very concerned that patients at risk for Type III PUHD can be easily awakened by severe, rapidly cycling hypoxemias (figure 5) or a room disturbance like a blood pressure check at night, become alert enough to be cognizant of their postoperative pain, then self medicate, fall back to sleep, and now drift to only seconds away from being found dead-in-bed. The fundamental concept that supports satisfactory "awake" sedation scores and ability to press a PCA button being sufficient to prevent narcotic induced sleep death is flawed. Favorable sedation scores while awake, or the requirement to press buttons while awake, do not protect patients from being at risk for death while asleep. Interestingly as an aside, the "discovery" of Type III PUHD as plausible cause for "Dead-in-Bed Syndrome" explains the often discounted statements made by many highly experienced, attentive nurses and physicians who in the past have had to defend themselves while under peer and malpractice review. Sadly, testimony asserting their postoperative patients were wide awake, completely alert with satisfactory sedation scores and asking for pain medication only minutes from being discovered dead, was rarely convincing.

Conventional threshold pulse oximeters are not capable of distinguishing discrete Type III signal patterns, either their cyclical desaturation clusters or the distinctive patterns of arousal failure. Their signal sampling, smoothing, and averaging algorithms prevent this high resolution capability. But they do alarm frequently from what they're able to process (a much less specific, collateral signal composite of merged and attenuated, real-time information). Derived through signal filtering, aka signal "smearing", these composite patterns unfortunately offer no interpretable details for risk assessment, as do our more distinct, high resolution patterns. Before our discovery of Type III PUHD, the incessant alarming introduced to the floors by this monitor was attributed to "false positive" triggering from signal noise and motion artifact. Regardless their origin, this posed an enormous nuisance to all clinicians forced to work through it. Those of us who fully appreciate the Type III pattern architecture, now see most of these "smeared" desaturation signals as instead being "true positives," meaning they're real...just too indistinct to reliably allow any differentiation between those reflecting milder, benign forms of sleep breathing disorders from others depicting the ominous sentinel patterns of cycling with arousal failure. We'll soon discuss in more detail what was to come from the inevitable clash between these legitimate but "smeared" Type III patterns and the huge disruptive element brought with them.

For now, let's revisit our fundamentals in summary. Standard threshold pulse oximetry on general care floors can serve only as a model to learn and deviate from if we're committed to detecting and distinguishing the three PUHD early. At best it's a selective late detector, made even later by our now being asked to reset its threshold to absurdly lower values, e.g. 80%. This new, industry supported, recommendation is being promoted as an "actionable threshold," perhaps because any sustained breach of such an extreme value would necessarily mandate resuscitation. However, resetting a pulse oximeter's threshold to 80% can only expose patients with Type I and II instability to further inaction and delay. All the recommendation really does is suppress the incessant alarming from "smeared" Type III patterns, the bulk of which self corrects through arousal. It's the industry's "Better late than never" monitoring mantra on steroids! From a business perspective, it markets the ability to monitor every patient on every floor while not being driven insane by constant alarming, and offers the reassurance that if you do monitor each and every patient, you won't be surprised by "Dead in Bed" events. But what's not told or even well understood is that you're essentially trading away these occasional "Dead in Bed" events for a considerably more common and costly critical care mortality and morbidity that further delays in Type I and II PUHD detection are sure to bring. We are suggesting that these recommended threshold modifications are more likely an industry's final (and somewhat desperate) attempt to preserve its now thirty year old threshold paradigm, perhaps unintentionally, but certainly inevitably exposing considerably more patients to further harm than can be possibly helped. Indeed, the industry's "Better late than never" mantra is a double edged scalpel, wounding far more than it heals when used to justify sustaining such an incapable status quo.

## Discussion

**Figure 6:**
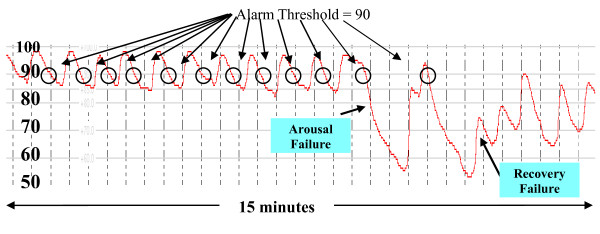
**"Alarm Fatigue" induced by the Type III pattern of figure 5**.

From the success it enjoyed in Operating Rooms and Specialty Care Units [90] came the assumption by the late 1990 s, albeit without much forethought, that threshold pulse oximetry (its policies included, e.g. 90% threshold) would seamlessly transition into the general care environment and culture without a wrinkle. However, significant problems were to surface immediately, the majority having little to do with issues of false security or obvious reductions in safety. Rather, these problems cut to the very heart of our human condition, our fundamental need for a sane working environment. Floor nurses found themselves immediately overwhelmed by incessant alarms blaring each time a resting patient began to cycle into their repetitive sleep apnea related desaturations. Figure 6 shows how threshold alarm processors reduce Type III patterns to states of "*Alarm on" OR "Alarm off"*.

These alarms were triggering so frequently, nurses were forced to come up with ingenious ways to control the disruption without disregarding policy. While their solutions were highly creative, they weren't always patient centric or in keeping with optimizing safety. One personal favorite is having the audible pulse tone and alarm on patient room oximeter dialed up to full volume, while the duplicate signals being sent off to the central nursing station are respectively muted and disarmed. Clever stuff, albeit a tad self serving, what people will come up with to preserve their sanity. Admittedly unfortunate for the room's occupant, their being kept awake all night, but certainly an effective way to curtail any possibility of disordered breathing while sleeping.

Because nothing was known about Type III patterns at the time and most patients were self rescuing through arousal anyway, the alarming problem was attributed to incidental patient movement and signal noise. Regardless, it was causing both widespread "alarm fatigue" (an already well recognized threat to patient safety) right from the start [91-94], and even worse for industry business...monitor abandonment. This drove industry leaders to begin looking for solutions through design improvements made to their motion attenuation filters in the oximetry sensors. While a separate issue and never a problem in the operating rooms, movement needed to be accounted for on hospital floors. Patient motion, especially from the finger where the sensor attached, could back then easily corrupt its signal and generate a false alarm. But this issue was just confounding the more important physiologic challenges being faced but not seen, the pervasive airflow reductions and associated desaturations that were occurring during sleep in the hospital.

Nevertheless, the prevailing view maintained that a new generation of "motion resistant" pulse oximetry would solve these problems [12,13], but then a large randomized prospective trial by Ochroch et al. completed at the University of Pennsylvania in 2006 [11] using threshold based, motion tolerant oximetry failed to show any clinical benefit overall, discrediting the "the false positive alarm premise" as the reason for its lack of efficacy. In addition, this trial made an interesting discovery; that threshold triggered pulse oximetry potentially has a dichotomous effect, helping some but adversely affecting others. This explains what is now called the "threshold oximetry paradox", a dichotomy where clinicians anecdotally observe that pulse oximetry can provide pivotal warnings for some patients, while randomized controlled clinical trials fail to reflect any overall benefit. The findings of this Penn trial suggest that these benefits to some patients were somehow being offset in others, so that in aggregate the threshold triggers were not helpful on the general floors studied.

In spite of the mounting, contrary evidence and all the initial disruption, our seduction by over-simplified threshold applications like standard pulse oximetry on the hospital floors continued. Many legitimate reasons can explain this, and none are meant to demean. There is a tendency for all of us to forget complex, abstract theory while immersed in moment to moment chaos as is so often the case in our hospitals. The statistical profiles of our monitoring and testing applications aren't easy concepts to grasp, and most of us can use regular reviews on the complexities represented by our PUHDs. This would include physicians as well as those dedicated clinicians most closely aligned to the hospital floor environments, our hard working floor nurses. They, quite honestly, were never trained to know these details we've been covering. Oximetry and its ill-fitting policy were largely forced on them. They, in return and without meaningful physician-led guidance, were compelled to modify their policies in ways that made sense to them, creating value through compromise. In order to assimilate pulse oximetry and not completely unravel their myriad workflow processes and policies that might otherwise endanger their patients, they created many interesting ways to work around its flaws. None of the references cited in this paper are even on their radar, and aren't suppose to be. In addition, few physicians understand themselves how this stuff works, or have the time to teach our nurses the information we've covered. Those physicians most likely to 'get it" are most often sequestered within their own unique environments like Critical Care Units, ORs, ERs, Post Anesthesia Care Units (PACU), and Sleep Laboratories. Critical Care Physicians are familiar with unstable patients because that's all they treat. Their patients start improving, wham, they're transferred to the floor, essentially disappearing. Anesthesiologists may be familiar with OR and PACU airway threats, but have scant experience with the floor events being discussed. ER Physicians are triage specialists, typically working tirelessly and quickly with some of the least sophisticated monitoring in the hospital, while Sleep Specialists are hidden away in environments furthest from our hospital floors. The monitoring industry has been the ongoing default educator for many of our nurses, and all business bias aside, from where do they get their clinical insight? Anesthesiologists and Critical Care Physicians...clinicians least familiar with the hospital floors' special needs.

This pervasive ineffectiveness contributes heavily to many ill-advised decisions, like the nascent movement toward "actionable thresholds" supported by the oximetry industry. Its "raising-the-threshold" technique (lowering the threshold value) on alarm triggers essentially remains untested in controlled, randomized trials to date, but our collection of solid clinical references regarding Types I, II, and III PUHDs should support your taking a justifiably skeptical position when asked to believe the best answer for detecting any serious clinical instability early is simply to select a more extreme threshold value, like an SPO2 of 80% or heart rates greater than 140 bpm [95]. Implementing extreme threshold values can certainly mitigate alarm fatigue, but any comfort provided by the silence will last only until the nurses, doctors, and families involved discover their patient near death.

These recently recommended modifications to SPO2 and heart rate thresholds expose the arbitrary and capricious provenance of all threshold values, and perhaps competency weaknesses of those making the recommendations. These thresholds are so readily modified because they were chosen as "best guesses" to begin with, without any analysis of the actual instability patterns generating their breaches. These new recommendations haven't been properly analyzed either. We've already discussed how our original (less extreme) thresholds are able to become reliable data/time fragments (achieving high sensitivity and specificity simultaneously), but when coupled with RRT activation in a DFGP, the overall process only capably detects and manages late instability leading to disappointing outcomes. Recall our beginning point. The highly complex and varied patho-physiologic patterns comprising unexpected instability in the general care environment simply can't be reduced to single, numeric threshold values capable of the early detection essential for successful rescues. Now the industry and its vested supporters are telling us to extend these arbitrary data/time fragments out even further, rendering them significantly less capable than they already are. Knowing people in the industry as we do, we understand their intentions are principled, but they're going at this the wrong way. They can't be blamed for scrambling to find solutions to the disruption problem, but these efforts reflect their significant lack of clinical understanding pertinent to the care being delivered on hospital floors. Blind, persisting loyalty to these threshold applications, and the threshold paradigm in general, is frankly both unsafe and dangerous.

An alternative effort to improve patient monitoring has focused on mathematically fusing multiple parametric values such as SPO2, heart rate, respiration rate, temperature, and in some cases etCO2 through statistical modeling, the composite then able to generate a variety of derivative indices [1,96], at once more statistically sound (reliable) and more capable. While this approach provides a larger and more robust data/time fragment and is an improvement, it remains an over-simplistic DFGP model, accordingly suffering from many of the same limitations as our traditional thresholds. It's becoming clear that more physiologic information must be synthesized to generate improved alarm systems and RRT triggers so that they can perform to optimal effect in this complex environment.

In response to these new realizations, the attention of the industry is now being redirected toward the development of a new class of patient monitor which, rather than alarming in response to simple threshold breaches, detects, identifies, quantifies and tracks the actual or conformational patterns of evolving death as they develop relationally across multiple parameters over time. An analogy in industry would be automotive traction control, where a computer detects and quantifies the relational pattern of a skid to provide an optimal response. A simplified example of this alternative approach can be envisioned by imagining a programmed patient monitor capable of detecting and tracking the relational geometric patterns drawn off the conventional, high resolution signals shown in figures 1, 2, 3. This would include an alarm processor programmed to automatically detect and quantify these patterns and identify the pattern types, finally tracking their severity over time so the response of any particular pattern to treatment can be monitored as well. Regardless the approach taken, there is general agreement that new and radical paradigms are required to engage the clinical complexities under discussion. The unique requirements for more patient-centered cognitive support systems means accepting that incremental advances in data fusion and statistical processing are insufficient [97]. The limitations of data/time fragment guided protocolization mandate that the processing engage the EMR so that all relevant data available can be included in the analysis going forward. The requirement for computational transparency, as well as the ability to process large parallel data streams across different scales in real time severely limit the options within our dominate technologic paradigms today [97]. Bottom line, the status quo and its derivatives get a generous C- in an industry where we all should insist on straight A's.

**Figure 7:**
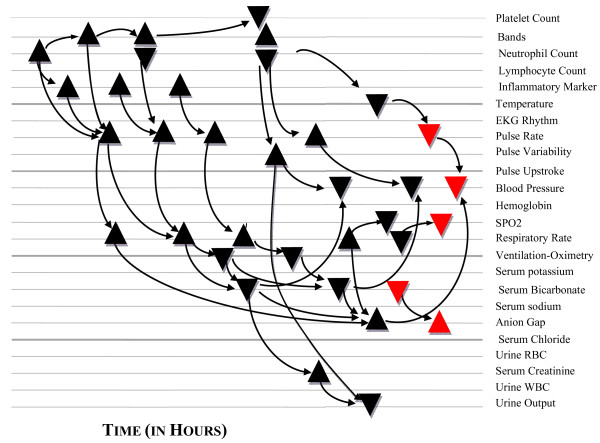
**Time Matrix of Relational Perturbations of Septic Shock**.

For those readers more technically inclined, Time Series Matrix Objectification (TSMO) provides one example of a radically new approach being developed. TSMO is a new hybrid signal processing technology capable of organizing and detecting patterns within large groupings of clinical parameters. Using this new technology, variations in these parameters (trends, perturbations, etc.) along parallel time-series (waveforms) are each converted into sequential and overlapping time domain objects of ascending complexity in a relational and inheritance based hierarchy. In this way, simple objects (such as a rise in white blood cell count) can be combined with other parallel objects (such as a relational rise in respiration rate, fall in platelet count, rise in pulse rate, fall in bicarbonate, rise in anion gap, etc.) to produce a complex and progressively enlarging two dimensional complex object or image comprised of smaller objects across the parallel waveforms of many parameters. The complex objects over the entire evolution of a sepsis cascade, for example, may be comprised of a very large and a progressively growing number of objects. Complex objects are assembled along a range of visual time scales and inherit all of the smaller objects from which they are derived, and can therefore be viewed and disassembled by the healthcare worker using touch screen interaction to provide complete real-time transparency. Using this technology, the pattern of undetected sepsis (a Type I PUHD), for example, begins with a focal rise or fall in white blood cell count or some other inflammatory marker, and then progresses over hours to days to involve increasing numbers of parallel parameters expanding over time. As shown in figure 7, this appears like a funnel cloud along the timed relational matrix of parallel patient parameters until final collapse occurs. Given the complexity of this Type I pattern, the futility of the application of data fragments such as any single threshold becomes clearly evident.

When objectified by the processor, these parallel object streams constructed from parallel clinical objects are now in a format searchable by an object search engine. Since clinical failures generally occur by specific mechanisms and/or along failure cascades, they will be comprised of object cascades across the objectified matrix producing definable complex objects (images), such as the complex cascade object of sepsis. The search engine can be programmed to automatically search the EMR and monitor data for these complex cascades at preselected intervals. In this way, rather than relying on traditional monitors in isolation, the entire EMR, including the relational outputs of the traditional monitors (if applied) are combined to produce a matrix of simple objects, and these simple objects are combined and searched to detect the more complex objects (images) which indicate the presence of a patho-physiologic cascade, e.g. PUHD I, II, and III.

Regardless the approach, and there are many alternatives, the broad goal [98] is to provide a highly sensitive and specific method of comprehensive data stream analysis with computational transparency so the healthcare workers can see the patterns when they've been detected and tracked. Presently there remains a strong residual focus on thresholds [2]. However, this is now changing rapidly and once our collective attention turns to PUHD detection, many alternative approaches will likely be developed. One of the purposes of this review is to encourage this development and the study of all new technologies with potential for detecting and tracking death patterns early.

## Conclusion

Some of you may still be puzzled by why patient monitors used in hospitals today have remained incapable of detecting and tracking the three common patterns of unexpected hospital death. We agree that this lack of progress in patient monitoring over the past decade is very difficult to explain, given our rapid advancements in other arguably less important technologies. For example, each year a new generation of smart phone is released, and it seems ironic that a patient can be dying of undetected sepsis while connected to a 21^st ^century monitor incapable of recognizing it early, while at the same time having a mobile phone in his pocket able to detect a song and its artist just by listening to it. The best explanation for the persistent and constrained focus of industry experts on our traditional monitoring technologies that would include our relentless searching for more "optimal" thresholds [2] is explained by the difficulty engaged by any science caught in its own expert paradigm.

Scientists despite their intellect, have a common human flaw which makes their behavior seem, at times, foolish. This flaw comprises a human trait to think as a herd, holding to common dogma, and rejecting opposing science, long after the dogma has reached the point of silliness to outsiders looking in. But there is good news. Since no professor wants to be caught clinging to old discredited science, when the dogma finally begins to break, this triggers a rapid abandonment of the old concepts and produces dramatic new directions of the science which often produces revolutionary benefits [98].

This year the FDA standards committee (ASTM) for pulse oximetry began to consider establishing minimum standards which would require that patient monitors marketed for use on the hospital general floor are capable of detecting and identifying at least the three common patterns of unexpected hospital death by 2014. However there remains no consensus and many committee members still advocate a continued search for the optimal alarm threshold. The standards (ASTM) group is open for membership (or input) to interested clinicians, patient safety advocates and researchers.

We began our discussion by taking a contrary position to what we identified as being conventional wisdom, thereafter exploring in detail three very distinct and irreducible pattern architectures associated (but not generally well understood) with unexpected clinical instabilities found in the general care environments of our hospitals. We explored as well the detection inadequacies of monitors currently available to us, and their impact on all patients forced to trust our opinions regarding their safety. We then called a question for your consideration, framed as follows: every competent clinician appreciates the lethality of sepsis and the importance of early detection for successful resuscitation...so how and why can it be argued reasonably, our setting threshold alarms for this disease at limits that would purposefully delay its detection?

We hope, now that you've taken the time to read this document and perhaps have supplemented your reading with our extensive references, that you'll agree there isn't an acceptable answer to the question above. Many of our routine practices are little more than culturally tenured, suboptimal approaches taken for granted for far too long. We're all fundamentally committed to excellence and have the talent to deliver it, but the forces we've allowed to shape the infrastructure of our professional lives are far more potent and pervasive than any of us can easily perceive. Add in our manic workloads and information inundation, and we all become pawns in a much grander scheme of healthcare mediocrity. We, your authors, like most healthcare workers, love complex information in our hectic clinical worlds to be "dumbed down" into appropriately understandable and reliable bits and bites...but we also recognize that the paradigm in which we now operate has gone too far with these reductionist processes. We're mired in a swamp of obsolete ideology and archaic tradition. It's continuing to cost lives needlessly and we need to be courageous enough to stand together and say it's time for change...it's the only way we can ever hope to help the millions of patients relying on us, our hospitals, and our monitors to keep them safe.

## List of abbreviations

RRT: (rapid response team); PUHD: (pattern of unexpected hospital death); RA: (respiratory alkalosis); MA: (metabolic Acidosis); RR: (respiratory rate); Ve: (minute ventilation); DFGP: (data- time fragment guided protocolization); EMR: (electronic medical records); TSMO: (time series matrix objectification);

## Competing interests

LAL holds patents and receives royalties relating to inventions in the field of patient monitoring and pattern detection.

JPC cites no conflicting interests.

## Authors' contributions

Both authors performed literature review, drafted, and approved the final manuscript.

## Authors' information

JPC is a senior staff anesthesiologist and past Chief of Staff of Hoag Memorial Hospital Presbyterian, Newport Beach, CA. He founded and is clinical advisor for the Hoag Rapid Response Initiative, has been a past member of clinical advisory boards for two leading oximetry companies, and is a clinical professor in the Department of Anesthesiology, UCLA David Geffen School of Medicine. LAL is a pulmonary and critical care physician and serves as executive director of the Sleep and Breathing Research Institute in Columbus Ohio. He also serves on the FDA standards committee for pulse oximetry monitoring.
